# Maintenance oral viscous budesonide reduces dilation recurrence after esophageal atresia repair: a prospective clinical study

**DOI:** 10.3389/fped.2026.1831346

**Published:** 2026-05-14

**Authors:** Cosimo Ruggiero, Giusy Russo, Denis Cozzi, Danila Volpe, Silvia Ceccanti, Paola Papoff, Miriam D’Orsi, Tonia Raso, Giulia Mancuso, Salvatore Oliva

**Affiliations:** 1 Pediatric Gastroenterology and Liver Unit, Maternal and Child Health Department, Sapienza – University of Rome, Rome, Italy; 2 Pediatric Surgery Unit, Maternal and Child Health Department, Sapienza – University of Rome, Rome, Italy; 3 Pediatric Intensive Care Unit, Maternal and Child Health Department, Sapienza – University of Rome, Rome, Italy

**Keywords:** anastomotic stricture, dilation, discontinuation effects, esophageal atresia, maintenance treatment, oral viscous budesonide

## Abstract

**Background:**

Recurrent anastomotic stricture (AS) remains a major cause of morbidity after esophageal atresia (EA) repair. Although oral viscous budesonide (OVB) reduces dilation requirements during active treatment, the durability of this benefit after treatment discontinuation remains unclear.

**Methods:**

We conducted a prospective, open-label, randomized study including children with repaired EA and steroid-responsive recurrent AS. Patients were randomized (1:1) to continue OVB maintenance therapy or discontinue treatment. The primary outcome was dilation-free survival. Secondary outcomes included dysphagia severity, radiologic stricture severity, and safety.

**Results:**

Twenty-two patients were included in the final analysis (OVB *n* = 12; control *n* = 10). At 6 months, dilation-free survival was significantly higher in the OVB group compared with controls (91% vs. 50%, *p* = 0.037). Dilation recurrence occurred in 25% vs. 70% of patients, respectively (*p* = 0.043). Median time to recurrence was longer with OVB (13 vs. 7 months). Maintenance OVB was independently associated with lower dilation risk (adjusted HR 5.68; 95% CI 1.08–27.4). Dysphagia scores were significantly lower in the OVB group, whereas radiologic severity showed only a trend toward improvement. No systemic adverse effects were observed.

**Conclusions:**

Maintenance OVB significantly prolongs dilation-free intervals in children with recurrent AS after EA repair, supporting topical anti-inflammatory therapy as a potential disease-modifying strategy.

## 1 Introduction

Anastomotic stricture (AS) represents one of the most frequent and clinically relevant complications following surgical repair of esophageal atresia (EA) (1), affecting up to 40% of patients during the first year of life. Recurrent strictures substantially impair feeding ability, growth, and quality of life, and often require repeated endoscopic dilations, exposing patients to procedural risks and healthcare burden (2–9).

Although mechanical dilation remains the cornerstone of treatment, AS recurrence is common, highlighting the need for disease-modifying strategies rather than purely mechanical approaches. Increasing evidence suggests that chronic mucosal inflammation contributes to fibrotic remodeling at the anastomotic site. Importantly, eosinophilic esophageal inflammation has been reported more frequently in patients with EA compared with the general pediatric population, suggesting a potential inflammatory-driven mechanism contributing to stricture persistence and recurrence (10, 11). Several pharmacologic strategies, including intralesional corticosteroids and mitomycin C, have been explored, but their efficacy remains limited and often requires repeated invasive procedures. Oral viscous budesonide (OVB) is an established topical anti-inflammatory therapy in eosinophilic esophagitis, achieving high rates of histologic and clinical remission with an excellent safety profile (12). Preliminary observational data suggest that OVB may reduce dilation requirements in EA-related AS during active treatment (13). However, whether this therapeutic benefit persists after treatment discontinuation remains unknown. Clarifying this aspect is essential to determine the potential role of OVB as a maintenance disease-modifying therapy.

The aim of this study was therefore to evaluate the sustained efficacy of OVB maintenance therapy compared with treatment discontinuation in children with repaired ED and steroid-responsive recurrent AS, focusing on dilation-free survival.

## 2 Methods

### 2.1 Study design and population

This prospective, open-label, randomized study was conducted at a tertiary pediatric referral center between January 2024 and June 2025. The study protocol was approved by the ethic committee (n. 6693-2023) and written informed consent was obtained from all participants' guardians.

Eligible participants were children aged 2–18 years with repaired EA and prior steroid-responsive recurrent AS, defined as absence of dilation requirements during 12 months of OVB therapy following at least three previous dilation sessions. Exclusion criteria included: previous intralesional esophageal steroid injection; additional esophageal surgery; diagnosis of eosinophilic esophagitis; neurological disorders affecting feeding; esophageal dilation within 12 months prior to enrolment; poor adherence to topical steroid therapy. Proton pump inhibitor (PPIs) therapy was permitted during the study.

Participant identifiable information will be collected as part of the source data and will be accessible only to authorized site personnel. Each participant will be assigned a unique identification number and referred to solely by this number during data analysis to ensure confidentiality.

### 2.2 Randomization and intervention

Patients were randomized 1:1 using stratified block randomization with sealed opaque envelopes.

Patients assigned to the intervention arm continued OVB therapy, whereas controls discontinued topical steroid treatment. OVB was self-prepared by mixing budesonide nebules with sodium alginate and administered orally at: 1 mg/day (<10 years) or 2 mg/day (≥10 years).

Patients were instructed to avoid eating, drinking, or rinsing for 30 min after administration.

### 2.3 Assessment of anastomotic stricture

Anastomotic stricture identification was based on clinic suspect and subsequent radiological and/or endoscopic confirmation. Clinical recurrence was assessed using the Dysphagia Symptom Score (DSS), a 4-point scale ranging from 0 (no dysphagia) to 4 (complete inability to swallow liquids) (13). Patients with a suspected anastomotic stricture (DSS >1) underwent barium swallow study and endoscopic evaluation.

Radiologic severity was assessed using the Stricture Index (SI): SI = [(*D*-*d*)/*D* × 100] where “*D*” represents the distal esophageal diameter and “*d*” the luminal diameter at the stricture site (14). In presence of radiological (SI > 50%) or endoscopic (inability to pass with a size caliber endoscope per age) evidence of anastomotic stricture, endoscopic dilation was performed for all patients enrolled. Endoscope-guided pneumatic dilation (EPD) was carried out by choosing a desirable diameter of balloon dilator, under sedation: the endoscopist passed the balloon catheter through the working channel and positioned at the mid-site across of the anastomotic stricture. A 2 mm step-up increasing diameter of 60 s each until reducing of tactile resistance was applied for a maximum of 3 times during the same session.

### 2.4 Follow-up

Patients underwent clinical evaluations at 1 month and every 3 months thereafter. Endoscopic and radiological assessment was performed every 6 months in all patients or when clinically indicated (DSS > 1). In case of new dilation patients were suggested to continue treatment or re-start topical steroid for patients in control group.

Adverse events related to both clinical and dilation procedures were recorded.

### 2.5 Outcomes

The primary endpoint was dilation-free survival and time to stricture recurrence. Secondary endpoints included: (1) Dysphagia severity; (2) Radiologic stricture severity; (3) Treatment safety.

### 2.6 Statistical analysis

Based on previous published data suggesting dilation risk <20% in treated patients by 6 months, and approximately 60% in untreated patients (13), a minimum sample size of 20 patients was estimated to achieve 80% power with *a* = 0.05.

Categorical variables were analyzed using Fisher's exact test. Continuous variables were analyzed using Mann–Whitney *U* test.

Event-free survival was assessed using the Kaplan–Meier curves and log-rank testing. Cox proportional hazards regression models evaluated predictors of dilation recurrence.

Statistical analyses were performed using SPSS v. 27 (IBM Corp., Armonk, N.Y., USA. All statistical tests were two-tailed, with a *p*-value < 0.05 considered statistically significant.

## 3 Results

### 3.1 Study population

**Figure 1:**
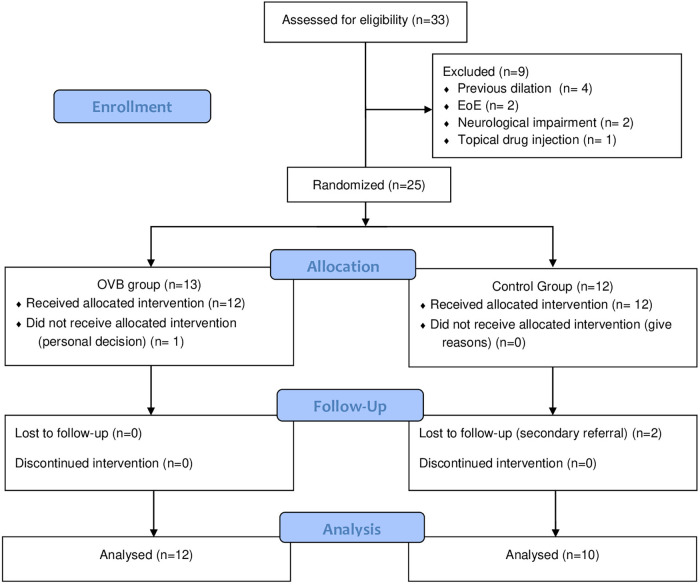
Flow diagram of study participants. EoE, eosinophilic esophagitis; OVB, oral viscous budesonide.

Among 33 patients screened, 25 patients were randomized and 22 completed follow-up and were included in the final analysis (OVB *n* = 12; control *n* = 10). One patient in the OVB group discontinued treatment for personal reasons, and two control patients were lost to follow-up due to referral to external centers (Figure 1). Baseline demographic and clinical characteristics were comparable between groups, except for higher PPIs use in the control group (Table 1). Anastomotic stricture dimensions, number and type of dilation regimen prior to OVB treatment are presented in Table 2.

**Table 1:** Demographic characteristic of patients in oral viscous budesonide (OVB) and control group.

Characteristic	Overall (*n* = 22)	OVB (*n* = 12)	Control (*n* = 10)	*p* value
Age (m), median (IQR)	41 (19–52)	43 (29–56)	37 (22–42)	0.33
Sex (M, F)	13, 9	8, 4	5, 5	0.07
Gestational age, *n* (%)				
Preterm	8 (36%)	5 (42%)	3 (30%)	0.44
Term	11 (50%)	5 (42%)	6 (60%)	0.1
Post-term	3 (14%)	2 (16%)	1 (10%)	0.27
Type of atresia, *n* (%)				
A	2 (9%)	2 (16%)	1 (10%)	0.93
B	-	-	-	
C	19 (91%)	10 (82%)	9 (90%)	0.89
Post-surgical complication, *n* (%)	9 (41%)	6 (50%)	3 (30%)	0.75
Early anastomosis stricture, *n* (%)	11 (50%)	7 (58%)	4 (40%)	0.07
Concomitant PPI, *n* (%)	11	3 (25%)	8 (80%)	0.039
Associated malformation, *n* (%)	15 (68%)	9 (75%)	6 (60%)	0.22
Cardiac defects	2 (9%)	1 (8%)	1 (10%)	-
VACTERL association	3	2 (16%)	1 (10%)	-
Treacher Collins syndrome	1 (4%)	-	1 (10%)	-
Microduplication Xp11.22p11.23	1 (4%)	-	1 (10%)	-
Kidney cyst	1 (4%)	1 (8%)	-	-
Duodenal atresia	3 (14%)	2 (16%)	1 (10%)	-
Brachydactyly	2 (9%)	2 (16%)	-	-

OVB, oral viscous budesonide; VACTERL, vertebrae, anus, heart, trachea, esophagus, kidney and limbs.

**Table 2:** Anastomotic stricture dimension, dilation sessions and regimen before OVB treatment.

OVB (*n* = 12)				Control (*n* = 10)			
AS (mm)	SI (value)	DS (n/y)	DR (PD/B)	AS (mm)	SI (value)	DS (n/y)	DR (PD/B)
8	0.6	11	PD	7	0.7	3	PD
8	0.7	4	PD	6	0.5	4	B
4	0.5	6	PD	8	0.7	5	PD
7	0.6	3	B	7	0.8	6	B
6	0.8	9	B	7	0.6	6	PD
7	0.8	8	PD	7	0.6	8	PD
4	0.6	6	B	4	0.8	4	B
7	0.8	6	PD	5	0.5	4	B
8	0.7	3	PD	6	0.7	9	PD
4	0.6	4	PD	6	0.5	12	PD
8	0.8	7	B	-	-	-	-
7	0.6	5	B	-	-	-	-

AS, anastomotic stricture (endoscopic measurement); SI, stricture index; DS, dilation session; DR, dilation regimen; PD, pneumatic dilation; B, Bougie (Savary–Gilliard).

### 3.2 Primary outcome

**Figure 2:**
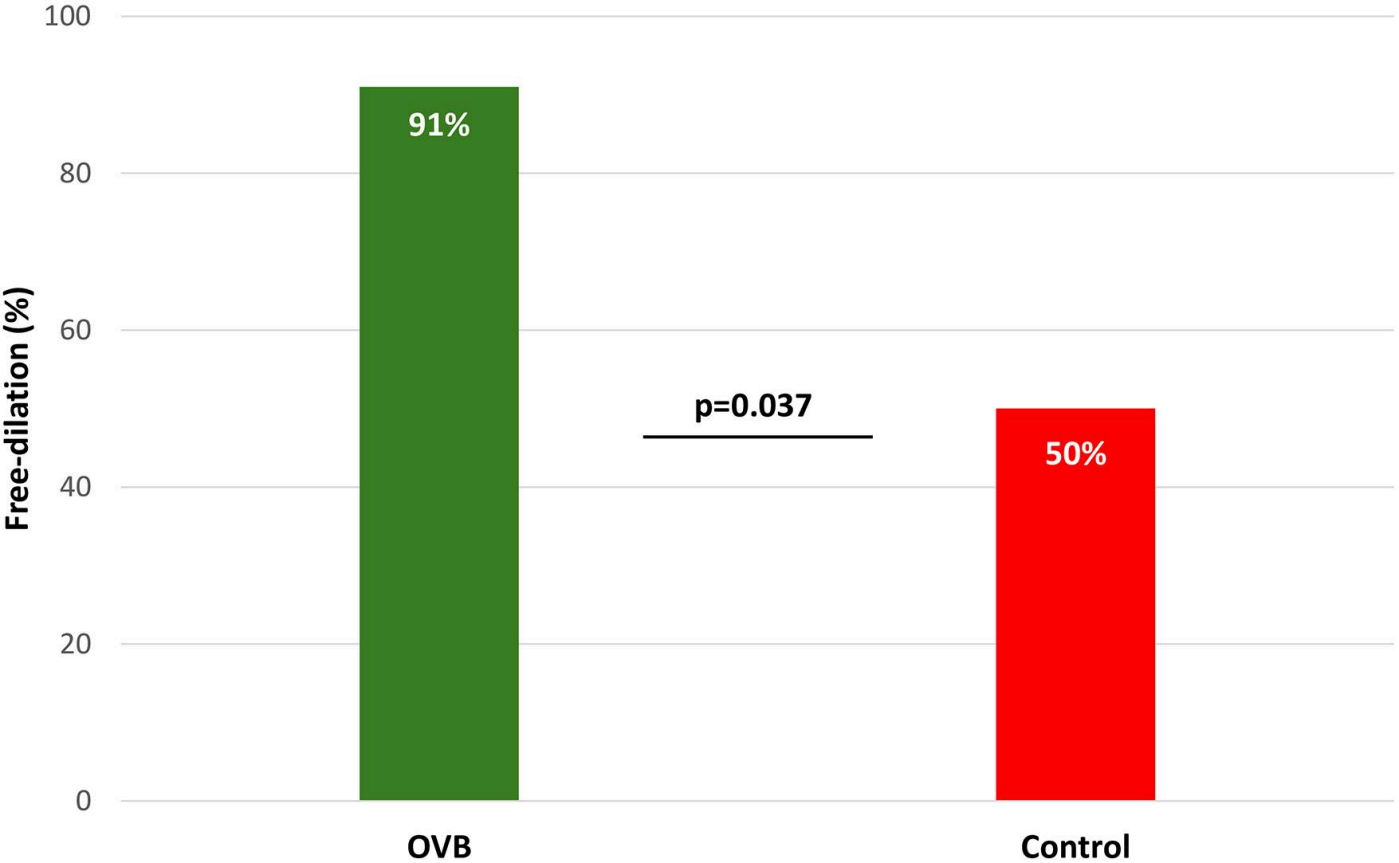
Free-dilation proportion in OVB and control group after 6 months. OVB, oral viscous budesonide.

Maintenance OVB significantly improved dilation-free survival. At 6 months, 11 of 12 patients (91%) in the OVB group remained dilation-free compared with 5 of 10 patients (50%) in the control group (*p* value = 0.037) (Figure 2). Overall dilation recurrence occurred in 3/12 patients (25%) receiving OVB and 7/10 patients (70%) in the control group (*p* = 0.043). Median time to recurrence was longer in the OVB group (13 months, IQR, 10–17) compared with controls (7, IQR, 3–9). The median number of dilation sessions required was 1 (range, 1–2) and 2 (range, 1–3) in intervention and control group, respectively.

**Figure 3:**
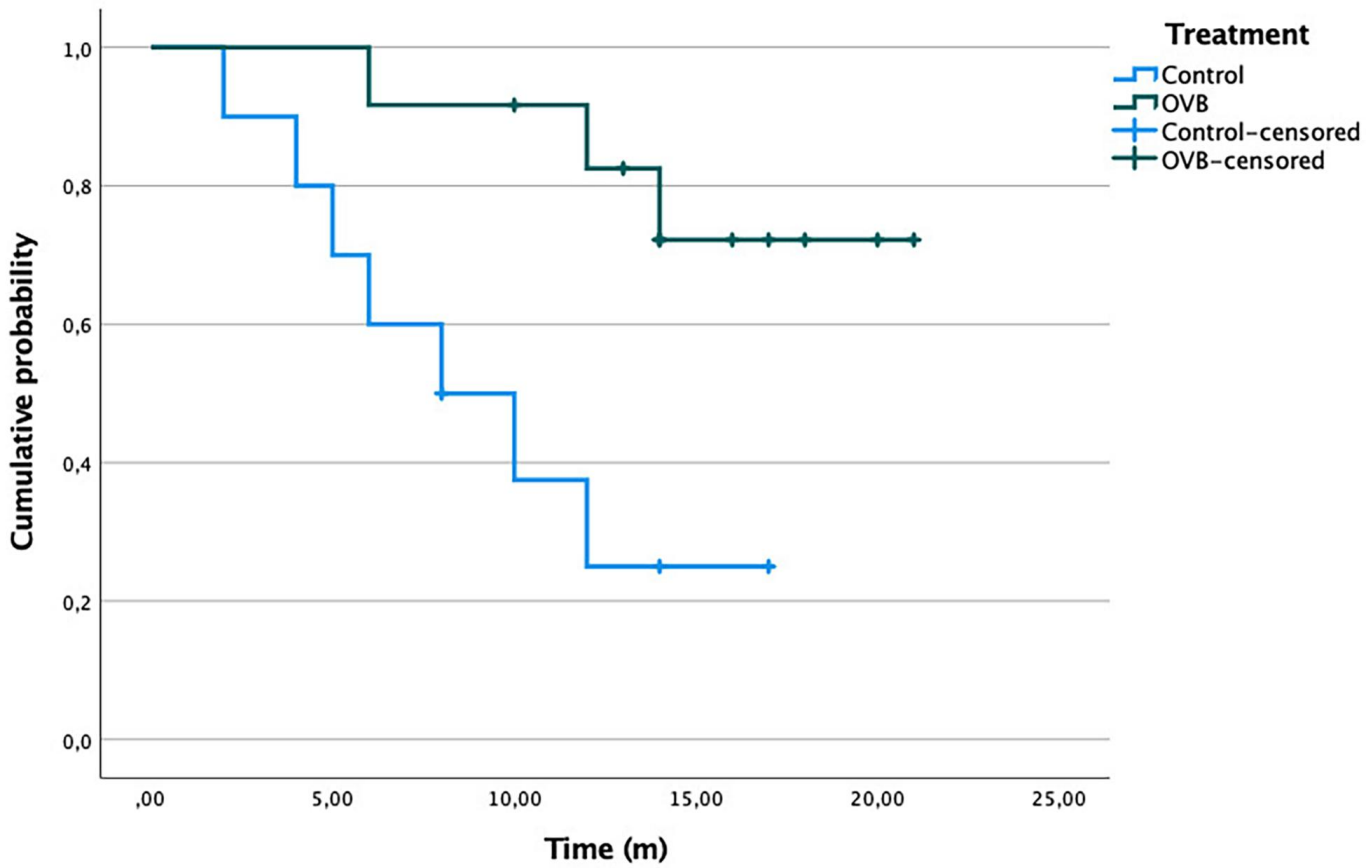
Kaplan–Meier curve for cumulative sustained time of free-dilation between groups. OVB, oral viscous budesonide; m, months.

Kaplan–Meier analysis confirmed significantly lower risk of dilation in the OVB group (log-rank: 0.03; Figure 3). In multivariable Cox regression adjusted for age, prematurity, number of prior dilations sessions, and early AS) demonstrated that maintenance OVB independently reduced dilation risk (HR 5.68 (95% CI, 1.08–27.4; *p* = 0.04; Table 3).

**Table 3:** Measures of treatment association through single and multivariate analysis.

Variables	Univariate			Multivariate		
	HR	CI, 95%	*p*	HR	CI, 95%	*p*
Treatment	5.03	1.27–19.6	0.02	5.68	1.08–27.4	0.04
Age	1.02	0.95–1.01	0.7	0.98	0.96–1.41	0.6
Sex	1.37	0.35–2.62	0.32	1.59	0.31–2.96	0.31
Early anastomosis	2.93	0.75–11.48	0.12	3.05	0.77–15.6	0.1
Post-surgical complication	1.64	0.45–5.94	0.44	1.86	0.84–4.27	0.26
Dilations	1.02	0.97–1.07	0.32	1.16	0.98–1.39	0.18

OR, odds ratio; HR, hazard ratio; CI, confidence interval.

### 3.3 Secondary outcomes

At 6 months, dysphagia severity was significantly lower in the OVB group than in controls (median DSS 0 vs. 2; *p* = 0.04). Radiologic severity, assessed by stricture index (SI), showed a trend toward lower values in the OVB group but did not reach statistical significance (0.41 [IQR 0.38–0.52] vs. 0.52 [IQR 0.42–0.56]; *p* = 0.063) (Table 4). No patient underwent dilation in the absence of clinical symptom recurrence.

**Table 4:** Clinical, endoscopic and radiological measurement at time of anastomotic stricture recurrence during the study period.

Measurements			
Patient	DSS	Endoscopy	SI
1	3	7	0.55
2	4	5	0.7
3	1	4	0.5
4	2	5	0.4
5	1	8	0.3
6	3	7	0.6
7	1	9	0.3
8	1	9	0.4
9	2	10	0.5
10	2	10	0.5

DSS, dysphagia symptom score; SI, stricture index.

**Figure 4:**
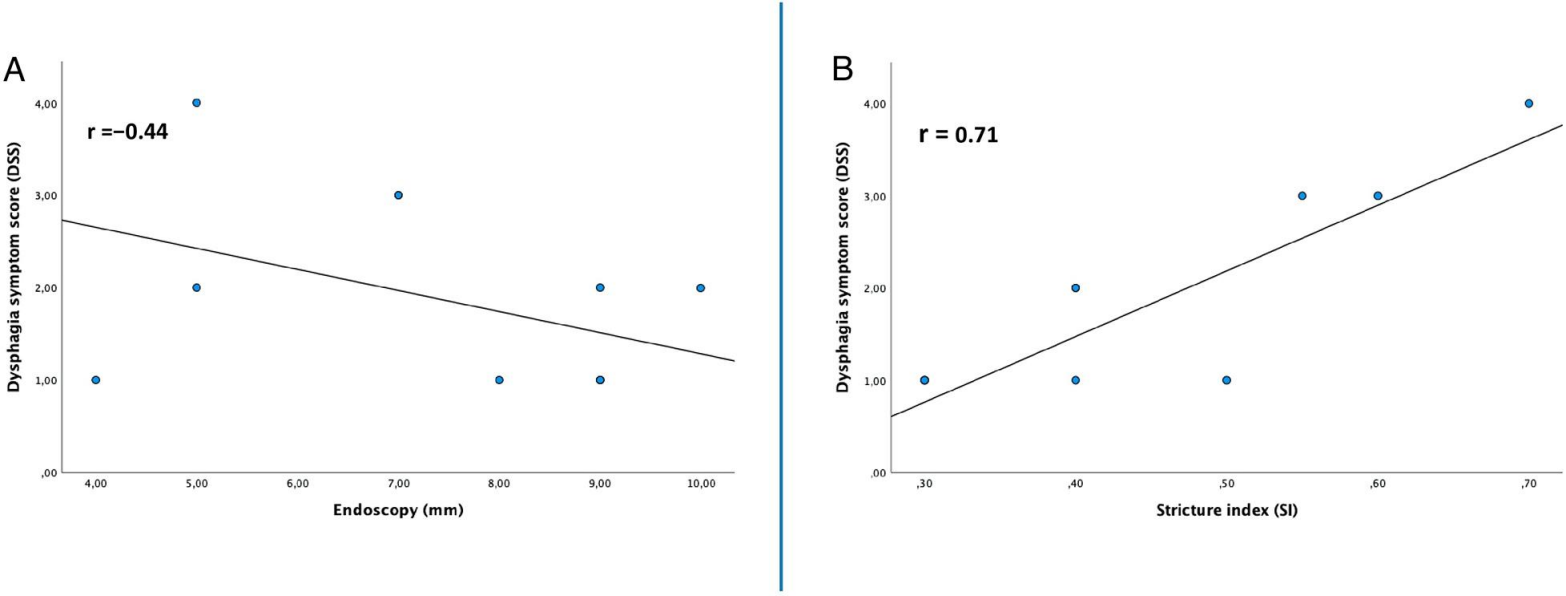
Correlation representation of clinic score (DSS), radiological index (SI) and endoscopic stricture at time of anastomotic stricture during the study period. DSS, dysphagia symptom score; SI, stricture index.

Symptom severity correlated strongly with endoscopic stricture severity (*r* = 0.71; *p* = 0.02) (Figure 4A) and showed a moderate inverse correlation with radiologic measurements (*r* = −0.44; *p* = 0.019) (Figure 4B).

Among patients with clinically significant dysphagia, endoscopic evidence of anastomotic stricture was observed in 8/10 (80%), whereas radiologic confirmation on barium swallow was present in 4/10 (40%). Two patients had dysphagia symptoms without endoscopic or radiologic evidence of luminal narrowing; both belonged to the control group and experienced symptom resolution after reintroduction of OVB.

Eight patients (45%) with early AS experienced dilation recurrence. Recurrence occurred in 1 patient in the OVB group and 5 patients in the control group, with earlier relapse in controls (median 2.5 months; IQR 1.75–3). Following dilation, patients in the control group restarted OVB based on prior clinical response and remained free of recurrent stricture over a median follow-up of 9 months (IQR 5.5–11).

No adverse events were recorded during dilation sessions or OVB treatment. Food impaction occurred in 3/10 (30%) control patients, and one required emergency endoscopic intervention. Serum cortisol levels remained within the normal range in all patients receiving topical steroids.

## 4 Discussion

This prospective, open-label randomized study demonstrates that maintenance OVB significantly prolongs dilation-free survival in children with recurrent AS after EA repair. At 6 months, patients receiving maintenance therapy showed a significantly lower recurrence rate compared with those who discontinued treatment. These findings highlight the clinical importance of continuing anti-inflammatory therapy over a prolonged period, given the substantial risk of AS recurrence following treatment withdrawal. Indeed, dilation risk after discontinuation was consistently higher compared with patients who remained on OVB therapy.

Importantly, most patients requiring dilation after treatment withdrawal were those who had previously presented with early postoperative AS. Although early AS did not emerge as an independent predictor in multivariable analysis, this observation suggests that early strictures may reflect a more aggressive inflammatory phenotype that could particularly benefit from prolonged maintenance therapy.

Compared with previous observational data (13), the proportion of patients remaining dilation-free after treatment discontinuation in our study was higher than that reported in cohorts that had never received OVB, suggesting that topical steroid therapy may exert a sustained anti-inflammatory effect even after withdrawal, potentially delaying fibrotic remodelling and reducing the need for subsequent dilation procedures.

The present study also provides important insights into the clinical monitoring of AS recurrence. The DSS identified the majority of patients with endoscopically confirmed AS, with 80% of symptomatic patients demonstrating luminal narrowing. Moreover, increasing DSS values were associated with progressive stenosis severity, as confirmed by both endoscopic and radiological measurements. These findings support the clinical relevance of symptom-based monitoring as an initial screening strategy. Moreover, no patients in clinical remission presented with anastomotic stricture during each 6-month follow-up. While this suggests the DSS is a reliable indicator of clinical assessment, its validity should be confirmed in larger cohorts.

Conversely, radiologic assessment showed lower sensitivity, with only 40% of symptomatic patients demonstrating radiologic confirmation of stenosis. Therefore, radiological evaluation alone should not be considered sufficient to guide dilation decisions. Nevertheless, radiologic imaging remains essential for defining stricture length, anatomical characteristics, and procedural planning for dilation techniques.

A subset of patients experienced dysphagia symptoms despite the absence of endoscopic or radiologic luminal narrowing. All these patients belonged to the control group and experienced symptom resolution following reintroduction of OVB therapy. This observation suggests that inflammation-driven functional impairment or secondary motility disorders may contribute to dysphagia in EA patients. Previous studies have demonstrated that dysphagia can occur in EA patients independently of structural stenosis in approximately 15%–50% of cases (15, 16). These findings raise the possibility that anti-inflammatory therapy could provide clinical benefit even in symptomatic patients without demonstrable anatomic narrowing, warranting further investigation.

The underlying pathophysiological mechanisms remain incompletely understood but likely involve locoregional inflammatory activity contributing to both fibrotic remodelling and esophageal motility dysfunction. In this context, maintenance OVB therapy may exert disease-modifying effects by targeting mucosal inflammation and preventing progression toward irreversible fibrosis.

The therapeutic benefit observed in this trial appears superior to previously reported pharmacologic approaches. Intralesional steroid therapy has demonstrated greater efficacy than mitomycin C in reducing AS recurrence (17, 18); however, these approaches typically require repeated invasive procedures and serial dilations, often with relatively short follow-up intervals (19, 20). In contrast, maintenance OVB provides a non-invasive therapeutic strategy with excellent tolerability and minimal systemic adverse effects (14, 21, 22). The favorable safety profile observed in this study is consistent with long-term experience using topical steroids in eosinophilic esophagitis (23).

Another relevant clinical implication concerns therapeutic strategy optimization. The increased recurrence observed after abrupt treatment discontinuation suggests that step-down approaches or gradual dose tapering may represent more appropriate strategies to maintain disease control while minimizing treatment burden. For this reason, closer endoscopic and/or radiological follow-up is recommended for patients who discontinue therapy, given the earlier recurrence of anastomotic stricture compared to patients on treatment. Further studies are required to define optimal treatment duration and tapering protocols.

Currently, there is no standardized clinical or instrumental algorithm for monitoring AS recurrence during follow-up. Furthermore, the relative diagnostic superiority of endoscopic vs. radiologic surveillance remains uncertain, as radiologic studies identify only a proportion of clinically relevant strictures (24). Our findings support an integrated monitoring strategy combining symptom assessment with targeted instrumental evaluation.

Several limitations of this study should be acknowledged. First, the trial was conducted in an open-label setting and included a relatively small sample size. Nevertheless, the randomized design and the magnitude of treatment effect strengthen the clinical relevance of our findings. Second, patients underwent different dilation techniques prior to enrollment; however, previous studies have not demonstrated significant differences in recurrence or complication rates between dilation modalities. Third, the pediatric population predominantly included younger children, limiting extrapolation to adolescents and adults. However, AS recurrence is most prevalent during early childhood, making this population particularly relevant for evaluating maintenance therapeutic strategies.

Future multicenter randomized studies including treatment-naïve cohorts are required to confirm these findings and to better define long-term safety and efficacy outcomes. Additionally, further investigation is needed to clarify whether maintenance therapy should be extended to symptomatic patients without structural stenosis and to determine whether dose-reduction strategies can maintain therapeutic benefit while improving adherence.

Overall, topical steroid maintenance therapy demonstrated superior ability to maintain dilation-free status compared with treatment discontinuation. These findings support the concept that sustained anti-inflammatory therapy may represent a disease-modifying strategy in EA-related anastomotic strictures.

## Data Availability

The raw data supporting the conclusions of this article will be made available by the authors, without undue reservation.
